# Endocrine-related osteoporosis: the state of the art

**DOI:** 10.3389/fendo.2026.1797546

**Published:** 2026-04-21

**Authors:** Iacopo Chiodini, Agostino Gaudio, Luigi Gennari, Domenico Rendina, Alfredo Scillitani, Fabio Vescini, Alberto Falchetti

**Affiliations:** 1 Department of Medical Biotechnology and Translational Medicine, University of Milan, Milan, Italy; 2 Department of Clinical and Experimental Medicine, University of Catania, Catania, Italy; 3 Department of Medicine, Surgery and Neurosciences, University of Siena, Siena, Italy; 4 Department of Clinical Medicine and Surgery, Federico II University, Naples, Italy; 5 Unit of Endocrinology, Ospedale “Casa Sollievo della Sofferenza”, San Giovanni Rotondo, Foggia, Italy; 6 Department of Oncology, University of Udine, Udine, Italy; 7 Endocrinology Unit, Azienda Sanitaria-Universitaria Friuli Centrale, P.O. Santa Maria della Misericordia, Udine, Italy

**Keywords:** FGF23-dependent phosphate-wasting disorders, hypercalciuria, hypercortisolism, male hypogonadism, primary aldosteronism, secondary osteoporosis, hyperparathyroidism

## Abstract

**Background:**

Endocrine-related secondary osteoporosis (ERSOP) comprises disorders in which bone fragility results from hormonal abnormalities other than age-related or menopausal hypogonadism. Its prevalence is underestimated, and timely recognition is essential because many forms improve or reverse when the underlying endocrine disturbance is corrected.

**Objective:**

This Review highlights the evolving concepts, unmet needs, and research opportunities in ERSOP, with focus on hypercortisolism, primary aldosteronism, male hypogonadism, hypercalciuria, and FGF23-dependent phosphate-wasting disorders.

**Recent findings:**

Mild autonomous cortisol secretion (MACS) is increasingly recognized as a contributor to fracture risk even at near-normal cortisol levels.

Primary aldosteronism promotes skeletal fragility via mineralocorticoid receptor activation, oxidative stress, hypercalciuria, and secondary hyperparathyroidism.

Male hypogonadism is a major driver of bone loss, and optimal fracture prevention may require combined hormonal and anti-osteoporotic therapy.

Differentiating PTH-dependent from PTH-independent hypercalciuria is crucial for management.

FGF23-mediated phosphate-wasting disorders impair mineralization and may respond to targeted therapy, including burosumab.

**Conclusion:**

ERSOP should be considered in patients with atypical osteoporosis phenotypes, including men, younger adults, and individuals with normal BMD but fragility fractures. Optimal care requires structured endocrine assessment, correction of the primary disorder, and integration with bone-specific therapy where appropriate.

## Introduction

Endocrine-related secondary osteoporosis (ERSOP) encompasses skeletal fragility resulting from hormonal diseases distinct from physiological aging and menopause. Despite its clinical relevance, ERSOP remains underdiagnosed, particularly in men, younger patients, and individuals who experience fractures despite normal bone mineral density (BMD). Unlike primary osteoporosis, many ERSOP forms are reversible if the underlying endocrine disturbance is identified and treated. However, recognition is often delayed due to inadequate screening and reliance on BMD alone, which does not capture alterations in bone quality.

Major endocrine causes of ERSOP include hypercortisolism, primary aldosteronism (PA), male hypogonadism (MH), hypercalciuria (HyC), and FGF23-mediated phosphate-wasting disorders. **Table 1** provides an overview of the most common conditions.

**Table 1:** Common endocrine causes of osteoporosis.

Condition	Mechanism	Clue
Hyperthyroidism	↑ Bone turnover (↑ resorption)	Weight loss, palpitations, tremor
Hyperparathyroidism	Cortical > trabecular bone loss	Hypercalcemia, kidney stones, hypercalciuria
Hypercortisolism	↓Bone formation, ↑/N resorption	Central obesity, striae rubrae, plethora, easy bruising, dorsocervical fat pad, proximal myopathy, unexplainable or resistant hypertension and/or diabetes,
Hypogonadism	↓Sex steroids → ↓bone mass	Amenorrhea, erectile dysfunction
Hyperaldosteronism	Associated long-term oxidative stress and chronic inflammation may increase osteoblast and osteocyte apoptosis, triggering abnormal bone metabolism, secondary OP, and fracture	Increased systemic blood pressure, normo-hypokalemia, secondary ↑ PTH by reduction of sodium and calcium re-absorption by the proximal renal tubules
Diabetes mellitus	Micro-architectural deficits	Neuropathy, retinopathy, normal BMD
GH deficiency	Reduced bone accrual/maintenance	Low IGF-1, short stature (if early-onset)
Mineral disorders Fibroblast growth factor 23 (FGF23)-related	- FGF23 endocrine, autocrine, and paracrine actions → renal phosphate leak with hypophosphatemia, inhibition of calcitriol biosynthesis, and bone loss.	↓Phosphate, ↓maximal tubular phosphate threshold normalized to glomerular filtration rate (TmP/GFR), ↑/↔ FGF23, ↑calciuria, nephrolithiasis

↓ = reduced; ↑ = increased; ↔ = normal; → = then it/they follow(s).

In this Review, we will briefly summarize some of the ERSOP causes still representing a challenge in their clinical identification and management: hypercortisolism, primary hyperaldosteronism, male hypogonadism, PTH-Dependent and PTH-Independent Hypercalciuria, and FGF23-related mineral disorders. Specific focus will be placed on new perspectives, diagnostic approaches, and therapeutic strategies for complex endocrine phenotypes associated with skeletal fragility. We conducted a comprehensive literature search of English-language articles published up to December 31, 2025, using PubMed and Google Scholar. The search included original clinical studies, systematic reviews, meta-analyses, and relevant guidelines, applying keywords related to endocrine disorders, mineral and bone metabolism, secondary osteoporosis, and skeletal outcomes. After screening and selection, the final analysis included two meta-analyses, six systematic re2views, 31 clinical studies, and two case reports or case series.

## Hypercortisolism

Both overt Cushing’s syndrome and mild autonomous cortisol secretion (MACS) are associated with trabecular deterioration and increased fracture risk. Notably, even high-normal cortisol levels or increased glucocorticoid receptor (GR) sensitivity can impair bone turnover. MACS, frequently detected as adrenal incidentalomas, and asymptomatic hypercortisolism is common, with a prevalence of 3% in individuals over 65 years old, particularly prevalent in patients with fragility fractures, where it has been reported to reach 17.6%. Identification of MACS through the “bone door” may allow early intervention, reducing both cardiovascular morbidity and fracture risk. However, guidelines on whom to screen among osteoporotic patients are still lacking. In addition to patients with MACS, even in healthy individuals, cortisol levels, though within normal ranges, could be associated with bone fragility. In fact, beyond cortisol concentrations, factors such as glucocorticoid receptor gene variants and altered activity of 11β-hydroxysteroid dehydrogenase enzymes contribute to skeletal fragility. Emerging evidence also implicates disrupted circadian cortisol rhythm in metabolic complications, though its impact on bone requires clarification. Indeed, cortisol levels and 11β-hydroxysteroid dehydrogenase activity have been suggested to be associated with bone mineral density in postmenopausal eucortisolemic women with osteoporosis (1), and cortisol sensitivity may influence vertebral fracture risk in eucortisolemic diabetic postmenopausal women (2, 3). Finally, several studies suggest that not only is the total daily production of cortisol important but also the rhythm of its secretion. Disrupting cortisol circadian rhythm may lead to cardiovascular complications and metabolic diseases (4). However, data on the effect of a disrupted circadian cortisol rhythm on bone health are not yet available. In the future, the idea of treating this “functional hypercortisolism” could perhaps be introduced with beneficial effects on bone health (5, 6).

## Primary aldosteronism

PA contributes to skeletal deterioration through mineralocorticoid receptor (MR)-mediated oxidative stress, renal calcium and magnesium loss, and secondary hyperparathyroidism. MRs have been identified in bone cells (7). Genome-wide association studies (GWAS) of multiple phenotypes associated with fractures showed an association with genes that cluster with pathways such as aldosterone signaling in epithelial cells (8). Animal models with PA demonstrate reduced bone strength and mineral content, reversible with spironolactone treatment (9, 10). Interestingly, a direct action of PTH on the adrenal gland and of aldosterone or angiotensin 2 on the parathyroid gland has been reported (11, 12). Clinically, PA is associated with lower BMD, impaired trabecular bone score, hypercalciuria, secondary hyperparathyroidism, and increased fracture risk than controls (13–18). In a recent large population-based matched cohort study from Sweden, patients with PA had an increased risk of hip fracture (19). A study using a longitudinal population data base from the Taiwan National Health Insurance showed PA patients have a higher risk of fractures than patients with essential hypertension (20), even though MR antagonist (MRA) treatment did not reduce osteoporotic fractures among female PA patients (20). On the other hand, in a case-control study among male veterans with heart failure, patients treated with MRA were significantly less likely to experience fracture compared to those without MRA treatment (21), but such a therapy gives discordant results on fracture prevention and osteoporosis treatment in PA patients (20, 21).

Moreover, glucocorticoid co-secretion, common in PA, may further exacerbate bone loss ()^22^. Thus, while MRA may reduce bone turnover markers, evidence for fracture prevention remains inconsistent. Conversely, adrenalectomy appears to reduce fracture risk in selected patients. PA should be considered in hypertensive patients with hypercalciuria, nephrolithiasis, or osteoporosis. “Finally, in a study on more than 300 individuals with and without osteoporosis, PA was observed in the 26.1% of patients with the concomitant presence of osteoporosis, hypertension and hypercalciuria, suggesting that PA should be considered among the causes of secondary OP (23, 24).”

## Male hypogonadism

MH remains a major contributor to ERSOP, due to deficits in androgens, estrogens, and muscle mass. MH also affects the function of various organs and quality of life (25–27), while broader definitions also include other testicular functions, such as the endocrine role of Sertoli cells and spermatogenesis (28). MH can also be classified based on the time of onset into congenital and acquired hypogonadism (29) and originates from central (30) or primary testicular failure. Prevalence varies due to inconsistent diagnostic criteria and methodological differences in testosterone measurement. Among the most frequent causes of MH (31) are a) Klinefelter syndrome, with an incidence of 9–22 cases per 10,000 male births; and b) Androgen deprivation therapy (ADT) in prostate cancer, the second most common cancer worldwide, making ADT a major potential cause of hypogonadism, which is estimated to affect 2–3% of males aged 55–70 years. Among the clinical sequelae of MH are reductions in BMD and increased bone remodeling, leading to rapid bone loss, particularly in the early stages of hormonal deficiency (32). Testosterone acts on bone both directly (by stimulating osteoblast proliferation and inhibiting apoptosis) and indirectly (by inhibiting osteoclast activity through stimulation of cytokine and insulin-like growth factor 1 secretion) (33). Thus, the bone loss results from decreased osteoblast activity, increased osteoclast activity, and reduced mechanical loading due to sarcopenia. Although testosterone is the primary male sex hormone, evidence highlights the role of estrogens in bone health. Individuals with aromatase deficiency (expressed in bone by fibroblasts and other cells) exhibit low BMD, which improves with estrogen therapy (32, 33). This rationale supports the use of selective estrogen receptor modulators (SERMs) in male osteoporosis, particularly in patients with low estrogen levels or those undergoing ADT (34). Another factor influencing the risk of osteoporotic fractures is sarcopenia, resulting from impaired testosterone stimulation. This leads to bone loss (due to reduced mechanical stress) and postural instability, which further increases the risk of falls (35). Finally, a study comparing BMD in patients with primary and central hypogonadism suggests a potential negative effect of FSH excess on the male bone mass, especially with the spine (36). Testosterone replacement therapy increases vertebral BMD modestly but lacks evidence for fracture risk reduction and carries potential risks, especially in older men (e.g., prostate cancer or cardiovascular events), which raises concerns about the long-term risk/benefit ratio (34). Thus, the efficacy of testosterone monotherapy in preventing bone loss, especially in elderly patients, remains uncertain, necessitating individualized assessment (37). Given these limitations, anti-osteoporotic drugs (with proven efficacy in maintaining BMD in males) remain essential in high-risk hypogonadal patients, including those on ADT. Current guidelines further recommend anti-osteoporotic treatment in high-fracture-risk patients (38), such as those receiving ADT for prostate cancer, for whom denosumab is indicated (39).

## PTH-dependent and PTH-independent hypercalciuria

Hypercalciuria (HyC) is characterized by a urinary calcium excretion above 250 mg/day in women and 300 mg/day in men on a standard calcium diet; alternatively, it can be diagnosed in individuals with urinary calcium excretion exceeding 4 mg/kg/day. In clinical practice, HyC is frequently associated with nephrolithiasis, osteoporosis, and metabolic bone disease, and it is strongly linked to low-trauma fractures, consequently requiring biochemical differentiation for appropriate management. It is typically classified as either PTH-dependent or PTH-independent, which has substantial diagnostic and therapeutic implications (40), and differentiating between PTH-dependent and PTH-independent forms is a critical issue. Thus, such a dichotomic classification is helpful to better describe the biochemical/clinical phenotype underlying HyC, and it may be useful to describe PTH-dependent HyC, a form of ERSOP, versus PTH-independent HyC.

### PTH-dependent HyC

In PTH-dependent HyC, increased calcium excretion in urine is directly caused by raised or improperly normal PTH activity. Two main forms of PTH-dependency can be distinguished in primary hyperparathyroidism (PHPT) and tertiary hyperparathyroidism (seen in the context of chronic kidney disease after long-standing secondary hyperparathyroidism) (41, 42). In both instances, PTH directly stimulates bone resorption and increases renal tubular calcium reabsorption. In addition, PTH enhances intestinal calcium absorption indirectly through the stimulation of 1,25(OH)_2_D_3_ synthesis. The increased filtered calcium load may exceed distal tubular reabsorptive capacity, resulting in hypercalciuria. PTH induces hypercalcemia by increasing intestinal calcium absorption and enhancing bone resorption (43).

### PTH-independent HyC

PTH-independent HyC is a heterogeneous condition and includes idiopathic hypercalciuria (IHyC), the most common form in the general population; it refers to situations characterized by increased urine calcium excretion that is not driven by elevated PTH levels. Its pathogenesis is multifactorial, alternatively involving increased intestinal calcium absorption, renal phosphate leak, enhanced bone resorption, or renal calcium leak (44–46). In IHyC, the serum calcium is normal and PTH is appropriately low or normal. It recurs frequently with a familial pattern, suggesting a genetic predisposition. Thus, a long-standing HyC, regardless of origin, necessitates targeted evaluation and management to prevent nephrolithiasis and skeletal complications. Other causes of PTH-independent HyC are summarized in **Table 2**.

**Table 2:** Causes of PTH-independent HyC.

Vitamin D intoxication (47)
Granulomatous diseases (e.g., sarcoidosis or tuberculosis) (48)
Thyrotoxicosis (49)
Immobilization (50)
Milk alkali syndrome (51)

Overall, persistent HyC, irrespective of PTH dependence, predisposes an individual to nephrolithiasis, nephrocalcinosis, reduced BMD, and increased fracture risk (especially in IHyC and PHPT) (44, 52). Thus, elucidating the underlying process is essential for focused management (53). IHyC management combines dietary measures and targeted pharmacotherapy. First-line non-pharmacologic measures include high fluid intake, e.g., a daily water intake greater than 3.5 L/day (46), to achieve large urine volumes, dietary salt restriction, and moderation of dietary protein while maintaining normal dietary calcium to avoid/limit bone loss. When lifestyle measures are insufficient or stones/recurrent hypercalciuria persist, thiazide or thiazide-like diuretics (e.g., hydrochlorothiazide or chlorthalidone) are the cornerstone therapy to reduce urinary calcium excretion and lower recurrence risk; potassium supplementation or potassium citrate is frequently co-prescribed to prevent thiazide-induced hypokalemia and/or to correct hypocitraturia, which itself increases stone risk. Treatment should be individualized after excluding secondary causes (e.g., hyperparathyroidism), with periodic monitoring of serum electrolytes, renal function, and urinary parameters (54–57).

## FGF23-related mineral disorders

FGF23-driven disorders reduce serum phosphate (PO) availability, hypophosphatemia (HypoPO), and calcitriol activation, impairing bone mineralization and increasing the risk of insufficiency fractures. Thus, PO is crucial for bone health and severe metabolic bone disorders, including osteoporosis, rickets, and osteomalacia, will result from PO chronic deficiency, i.e., chronic HypoPO (58, 59). Chronic HypoPO is usually defined as severe serum PO levels < 0.8 mMol/L (< 2.5 mg/dl) in adults (60). PO homeostasis is tightly regulated through the functional interaction between at least four organs/apparats and three hormones, namely the parathyroids, gut, kidneys, and skeleton on the one side and vitamin D biological system, PTH, and FGF23 on the other (61). Considering the high PO biodisponibility, PO homeostasis is largely regulated in the kidney and, usually, chronic HypoPO is caused by a reduced tubular reabsorption of phosphate from the glomerular filtrate, i.e., renal phosphate leak RPhL (62). RPhL may be PTH-related or non-PTH-related (61); the latter is typically caused by elevated or inappropriately normal FGF23.

## Mineral disorders related to FGF23

PO is one of the six chemical elements essential for life on Earth. It plays pivotal roles in various vital processes, including energy metabolism, protein biosynthesis, acid–base balance, and cellular membrane integrity (58). PO is also crucial for bone health and several metabolic bone disorders, including osteoporosis, rickets, and osteomalacia, can be caused by PO chronic deficiency, i.e., chronic hypophosphatemia (hypoPO) (59).

Chronic hypoPO is usually defined as severe serum PO levels < 0.8 mMol/L (2.5 mg/dl) in adults (60). RPhL may be PTH-related (see relative paragraph) or non-PTH-related (60). RPhL not-PTH-related is usually caused by a dysregulation of FGF23 endocrine activities (63).

FGF23 is a bone-hormone produced by osteocytes which, contextually, reduces the tubular expression of sodium-phosphate co-transporter type 2A and 2c (NaPi-IIa and IIc), the major player of tubular PO reabsorption, and inhibits the 25-hydroxyvitamin D 1-alpha-hydroxylase, which catalyzes the hydroxylation of calcifediol to calcitriol. Both these FGF23 endocrine actions are Klotho-mediated (64). A significant percentage of patients with RPhL not-PTH-related show elevated or inappropriately normal FGF23 levels related to PO serum levels (65). A FGF23 functional allelic variant, *FGF23^C716T^*, which induces the biosynthesis of a protein with a threonine-methionine amino acid change at 239 position, FGF23^T239M^, is characterized by a prolonged half-life and increased affinity to FGFR. The FGF23^T239M^ variant is overexpressed in RPhL not-PTH-related subjects and is associated with a reduced bone mass peak (66, 67). Also, FGF23 paracrine and autocrine actions directly influence bone turnover and homeostasis. In experimental models, FGF23 shows a bimodal autocrine-paracrine effect on osteoblast and bone mineralization. While FGF23 promotes bone mineralization under physiological concentration, it suppresses both osteoblast differentiation and bone matrix mineralization in supraphysiological concentration. This latter activity appears independent of klotho and FGF23 systemic effects on PO homeostasis.

A RPhL FGF23-related must be considered in patients with chronic hypoPO (64, 68). In these patients, first-line biochemical tests must include determination of serum levels of calcium, albumin, PO, magnesium, PTH, alkaline phosphatase, calcifediol, creatinine, and of urinary levels of PO and creatinine. Using serum and urinary levels of PO and creatinine, the maximal tubular phosphate threshold normalized to glomerular filtration rate (TmP/GFR) could be quantified. The RPhL not-PTH-related is characterized by hypoPO, low TmP/GFR, calcium, magnesium, PTH, and calcifediol within the normal range. This clinical condition is usually caused by increased levels of FGF23 or, more rarely, of other phosphatonins (64). When RPhL is clinically evident, FGF23 levels inappropriately detectable considering the hypoPO or, in any case, ≥30 pg/ml are diagnostic for a RPhL-FGF23-related (65, 69). Its optimal treatment is debated. Vitamin D replacement or supplementation with its inactive form, according to local guidelines, oral PO salts, and active vitamin D hydroxylated analogues (e.g., calcitriol) is the conventional therapy. In patients with RPhL-FGF23-related and FGF23 levels ≥100 pg/ml, a treatment with burosumab, a recombinant human monoclonal antibody IgG1 against FGF23, should be considered in the clinical suspicion of a tumor-induced osteomalacia (TIO), at least in the USA and Italy (70, 71). TIO is a paraneoplastic syndrome, usually caused by small, benign, and slow-growing phosphaturic mesenchymal tumors (PMTs). Clinically, TIO is characterized by RPhL causing reduced BMD and osteomalacia (72). Treatment with oral PO salts and active vitamin D analogues should be discontinued seven days prior to burosumab treatment in TIO patients (72).

## Clinical implications

ERSOP should be proactively suspected in several cases: a) eugonadal men and premenopausal women with fragility fractures, b) individuals with fractures despite normal BMD, c) hypertensive patients with HyC or nephrolithiasis, d) patients with adrenal incidentalomas with suspected MACS, e) patients receiving treatments with recognized skeletal toxicity, and f) individuals with symptoms or laboratory evidence of endocrinopathy. **Table 3** provides several factors suggestive of ERSOP.

**Table 3:** Clues (diagnostic indicators and suspicion triggers).

**1. Atypical Patient Profile:**
• Young men or premenopausal women with fragility fractures.
• Fractures without significant trauma or minimal bone loss on imaging
**2. Clinical Red Flags:**
• Unexplained weight changes, fatigue, or altered libido.
• Persistent bone pain or muscle weakness.
**3. Laboratory Abnormalities:**
• Hypercalcemia, hypocalcemia, or hypercalciuria
• Abnormal thyroid, cortisol after Dex-suppression, aldosterone, or sex hormone levels.
• Elevated PTH
• ↓Phosphate, ↓TmP/GFR, ↔ calcemia, 25OHD, 1,25(OH)_2_D and PTH, ↑/↔ FGF23
**4. Specific Endocrine Disorders:**
• Hyperthyroidism – Increased bone turnover.
• Hyperparathyroidism – Cortical bone loss - hypercalciuria.
• Cushing’s Syndrome – Glucocorticoid excess reduces bone formation.
• Primary hyperaldosteronism – Increased osteoblast and osteocyte apoptosis, triggering abnormal bone metabolism.
• Hypogonadism – Especially in men and young women.
• Diabetes Mellitus – Altered bone quality despite normal BMD.
• GH/IGF-1 deficiency – Affects peak bone mass and maintenance.
• ↑/↔ FGF23 – Renal phosphate leak and bone loss.
• Hypercalciuria – Affects bone mass peak and maintenance
**5. Medications/History:**
• Long-term glucocorticoids.
• Aromatase inhibitors or androgen deprivation therapy.
• History of pituitary tumors, thyroid disease, or adrenal disorders.
• Parenteral iron (polymaltose) administration or antiretroviral drugs.
**6. Specific signs and symptoms of endocrine disorders** See as reported in **Table 1**.

↓ = reduced; ↑ = increased; ↔ = normal; TmP, tubular maximum reabsorption of phosphate; GFR, glomerular filtration rate; Dex, dexamethasone. Bold values indicate clinically relevant abnormalities that should prompt further evaluation for secondary/endocrine causes of bone fragility.

A practical list of red flags includes a) elevated 24hrs urinary free cortisol and/or elevated late night salivary cortisol and/or not-suppressible cortisol level after dexamethasone suppression test; b) elevated aldosterone-to-renin ratio; c) low T ± low estradiol; d) HyC with normal or high PTH; and e) low PO serum levels with elevated/inappropriate FGF23.

Common pitfalls include under-recognition of endocrine causes, over-reliance on BMD, inadequate hormonal screening, and insufficiently tailored therapy, indicating gaps in both in the basic knowledge of the problem represented by ERSOP and in its clinical management, which can determine a missed diagnosis and/or an inadequate pharmacological treatment of forms of ERSOP, as summarized in **Table 4**.

**Table 4:** Current pitfalls in diagnosis and management.

**1. Under-recognition of secondary causes:**
• Especially in men and younger patients.
• Osteoporosis is often misclassified as primary due to age bias.
**2. Over-reliance on BMD alone:**
• Many endocrine disorders affect bone quality without major BMD changes.
• Fragility fractures can occur at “normal” BMD levels.
**3. Inadequate screening for endocrinopathies:**
• Failure to check hormone panels or investigate metabolic causes in unusual clinical settings.
**4. Delayed or missed diagnosis:**
• Symptoms of underlying endocrinopathies (like fatigue, mood changes, or metabolic changes) often go unexplored.
**5. Sub-optimal treatment:**
• Using bisphosphonates alone without correcting the hormonal imbalance.
• Lack of an interdisciplinary approach (*e.g*., endocrinology input).
**6. Limited awareness of drug-induced osteoporosis:**
• Steroids, antiepileptics, SSRIs, parenteral iron (polymaltose), antiretroviral, and antidiabetics may be overlooked as culprits.
**7. Systemic symptoms:**
• Weight gain, truncal obesity, myopathy, fatigue, polyuria, or low libido.

Bold values indicate clinically relevant abnormalities that should prompt further evaluation for secondary/endocrine causes of bone fragility.

In clinical practice, correction of the underlying endocrine disorder should be prioritized whenever it is potentially reversible and not immediately associated with very high fracture risk. For example, adrenalectomy in MACS, parathyroidectomy in PHPT, treatment of primary aldosteronism, or testosterone replacement in carefully selected hypogonadal men may substantially improve skeletal outcomes over time. However, in patients at very high fracture risk, such as those with recent vertebral fractures, hip fractures, DXA T-score ≤ −2.5 SD with additional risk factors, or multiple fragility fractures, initiation of anti-osteoporotic therapy should not be delayed. In these cases, endocrine-targeted treatment and bone-specific therapy should be started concomitantly to rapidly reduce fracture risk. This integrated approach reflects the need to balance pathophysiological correction with immediate fracture prevention.

**Figure 1:**
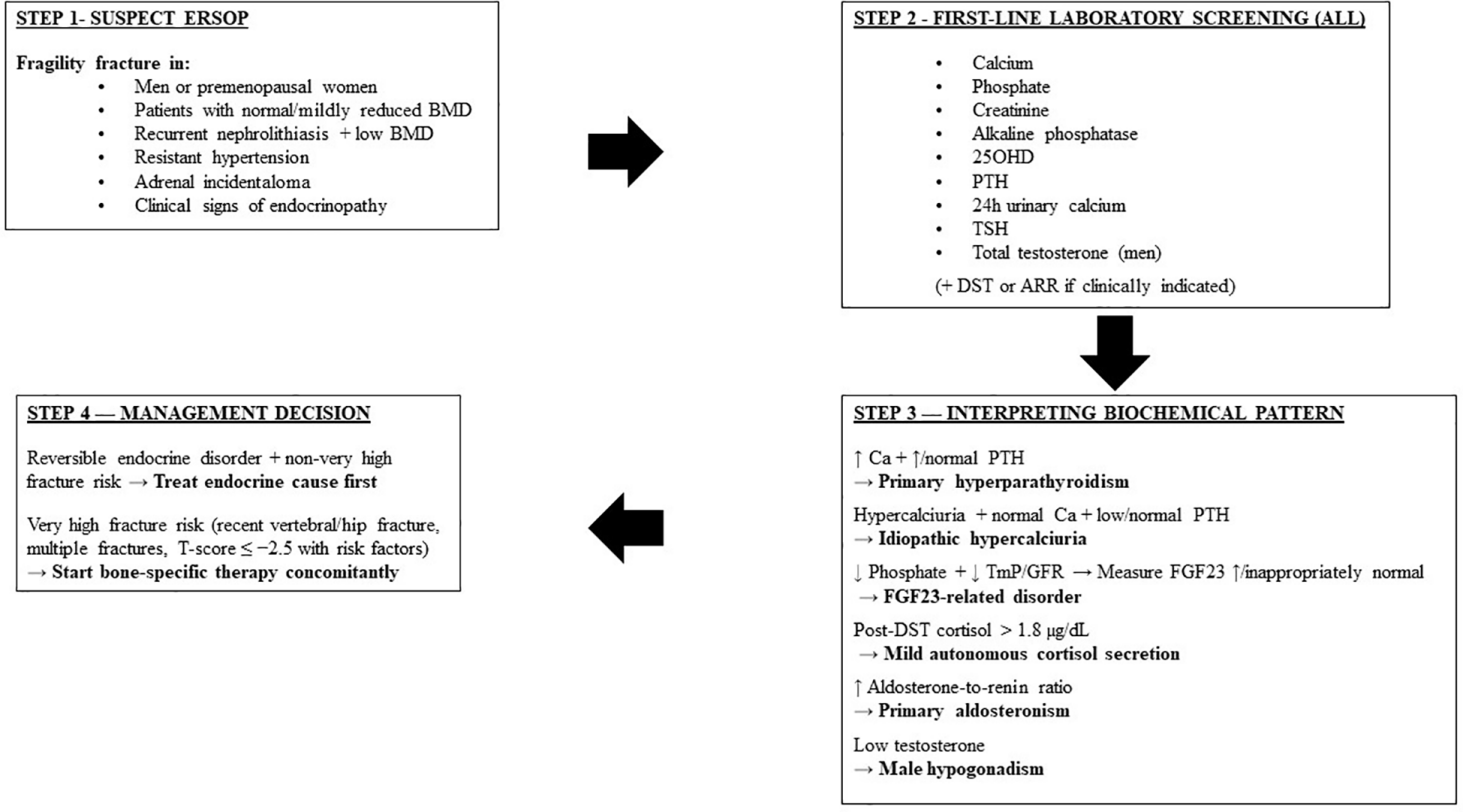
Flowchart for the diagnostic approach to suspected endocrine-related secondary osteoporosis (ERSOP).

To facilitate translation of these concepts into clinical practice, we propose a pragmatic stepwise diagnostic workflow for patients with suspected endocrine-related secondary osteoporosis (**Figure 1**). This flowchart integrates clinical suspicion triggers, first-line laboratory screening, and pattern-based targeted endocrine investigations, aiming to support timely identification of reversible causes of skeletal fragility.

## Conclusions

A more accurate diagnosis of endocrine-related skeletal disorders relies on combining several complementary approaches, such as detailed clinical evaluation, comprehensive systematic hormonal profiling, measurement of bone turnover markers, dual-energy X-ray absorptiometry (DXA), and advanced imaging techniques such as the DXA-related trabecular bone score (TBS) and high-resolution peripheral quantitative CT (HR-pQCT). If these elements were integrated, they would provide a much clearer picture of bone quality and the underlying mechanisms driving skeletal fragility.

Improving patient care requires a structured and multifaceted strategy. This typically includes targeted treatment of the primary endocrine disorder and, when necessary, the addition of specific anti-osteoporotic medications. In selected patients, interventions such as adrenalectomy, receptor antagonists, testosterone replacement, phosphate supplementation, vitamin D analogues, or burosumab can lead to substantial improvements in bone health.

Looking ahead, several key areas still need clarification. Increasing attention is being paid to bone quality rather than quantity alone, highlighting the importance of micro-architectural integrity beyond standard BMD measurements. It is also becoming clear that current fracture-risk tools like FRAX may underestimate risk in secondary forms of osteoporosis (73). Meanwhile, biomarkers of bone turnover are emerging as valuable tools for both diagnosis and monitoring treatment responses (72).

Future research priorities include conducting prospective studies that evaluate fracture outcomes specifically in ERSOP, defining genotype–phenotype relationships in conditions such as MACS, PA, MH, HyC, and FGF23-related disorders, and integrating imaging data, biomarkers, and machine-learning approaches to enable more personalized risk prediction. Another important line of investigation will explore endocrine-targeted therapies capable of modulating cortisol exposure, mineralocorticoid receptor activation, or phosphate homeostasis.

Ultimately, the greatest challenge and opportunity is to shift clinical practice from treating osteoporosis as an isolated condition to addressing the endocrine disturbance that underlies the bone disease. In essence, the core principle can be summarized in a single aphorism: “Treat the endocrine disorder first, and osteoporosis will often improve.”
